# *In vivo* evaluation of the synthesized novel 2-benzyloxybenzaldehyde analog CCY-1a-E2 for the treatment of leukemia in the BALB/c mouse WEHI-3 allograft model

**DOI:** 10.3892/ol.2012.1079

**Published:** 2012-12-17

**Authors:** CHINGJU LIN, JAI-SING YANG, SHIH-CHANG TSAI, CHIN-FEN LIN, MIAU-RONG LEE

**Affiliations:** 1 Departments of Physiology, China Medical University, Taichung 40402, Taiwan, R.O.C.; 2 Pharmacology, China Medical University, Taichung 40402, Taiwan, R.O.C.; 3 Biological Science and Technology, China Medical University, Taichung 40402, Taiwan, R.O.C.; 4 Biochemistry, China Medical University, Taichung 40402, Taiwan, R.O.C.

**Keywords:** 2-benzyloxybenzaldehyde, CCY-1a-E2, leukemia WEHI-3 cells, BALB/c mouse WEHI-3 allograft model, safety evaluation

## Abstract

Our previous study demonstrated that the 2-benzyloxybenzaldehyde analog CCY-1a-E2 is a potent compound against HL-60 human leukemia cell lines. To investigate the potential therapeutic application of CCY-1a-E2 for leukemia, we analyzed the antileukemic effects and safety of CCY-1a-E2 in the BALB/c mouse WEHI-3 allograft model. Our results showed that CCY-1a-E2 decreased the percentage of viable cells in a concentration-dependent manner. The IC_50_ of CCY-1a-E2 was 5 *μ*M for the 24-h treatment of WEHI-3 cells. We examined the antileukemic activity of CCY-1a-E2 in the BALB/c mouse WEHI-3 allograft model. The CCY-1a-E2 (100 mg/kg) group was not found to have significantly decreased body weight compared with the control group, while the leukemia group was found to have significantly decreased body weight compared with the control mice. The CCY-1a-E2 (100 mg/kg) group showed no difference in spleen and liver weight, but significantly decreased levels of CD11b and CD45 compared with the leukemia group. In safety evaluation analysis, CCY-1a-E2 had no adverse effects on renal, hepatic and hematological parameters. Based on these observations, CCY-1a-E2 has efficacious antileukemic activity in the BALB/c mouse WEHI-3 allograft model.

## Introduction

Leukemia is a malignant cancer in humans (1). The characteristics of leukemia include uncontrolled cell growth and disrupted differentiation of hematopoietic cells (2,3). In Taiwan, 3 per 100,000 individuals succumbed to leukemia in 2011 according to the Department of Health, Executive Yuan, R.O.C. (Taiwan; http://www.doh.gov.tw/EN2006/). The clinical therapies for leukemia include chemotherapy, radiation and bone marrow transplant (4–6). However, these strategies have not been shown to be satisfactory for the treatment of leukemia, which has led to researchers focusing on the discovery of new compounds.

**Figure 1:**
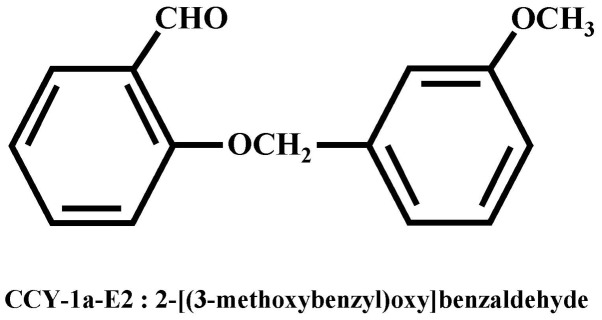
Chemical structure of CCY-1a-E2.

Benzyloxybenzaldehyde derivatives are known for their multiple biological effects, including antimicrobial infection (7), anti-inflammatory effects (8–10), phospholipase D (PLD) inhibition (10), neutrophil superoxide anion degeneration (11), adenylyl cyclase activation (8) and anticancer activities (12). In recent years, we have designed and synthesized a new series of 2-benzyloxybenzaldehyde derivatives as potential antileukemic agents (12). Our previous study demonstrated that the 2-benzyloxybenzaldehyde analog CCY-1a-E2 (2-[(3-methoxybenzyl)oxy]benzaldehyde) (Fig. 1) is a potent compound against HL-60 leukemia cells *in vitro*(12). CCY-1a-E2 induced G_2_/M phase arrest and induced cell apoptosis in HL-60 cells (12). However, the cytotoxic effects of CCY-1a-E2 on WEHI-3 leukemia cells and the antileukemic activity *in vivo* have not been fully clarified. In the present study, we demonstrated that CCY-1a-E2 induced growth inhibitory effects in WEHI-3 leukemia cells *in vitro* and *in vivo*.

## Materials and methods

### Chemicals and reagents

Dimethyl sulfoxide (DMSO) was obtained from Sigma-Aldrich Corp. (St. Louis, MO, USA). RPMI-1640 medium, penicillin-streptomycin, trypsin-EDTA, fetal bovine serum (FBS) and L-glutamine were obtained from Gibco/Life Technologies (Carlsbad, CA, USA). The FITC-labeled anti-mouse CD3, PE-labeled anti-mouse CD19, FITC-labeled anti-mouse CD11b and PE-labeled anti-mouse Mac-3 antibodies were obtained from BD Pharmingen Inc. (San Diego, CA, USA).

### Cell culture

The WEHI-3 murine myelomonocytic leukemia cell line was purchased from the Food Industry Research and Development Institute (Hsinchu, Taiwan). Cells were maintained in RPMI-1640 medium supplemented with 10% FBS, 2 mM L-glutamine, 100 U/ml penicillin and 100 *μ*g/ml streptomycin at 37°C in a humidified atmosphere of 5% CO_2_(13).

### Viability determination

The 3-(4,5-dimethylthiazol-2-yl)-2,5-diphenyltetrazolium bromide (MTT) assay was performed to determine the cell proliferation of CCY-1a-E2-treated WEHI-3 cells. WEHI-3 cells (∼2×10^4^/well) were placed into 96-well plates for 24 h. CCY-1a-E2 was dissolved in DMSO then individually added to the wells at final concentrations of 0.78, 1.56, 3.13, 6.25, 12.5 and 25 *μ*M, and 0.1% of DMSO in culture medium was added to the well as the control group. Following treatment for 24 h, cells from each well were harvested for the determination of viability using an MTT method as described previously (14). The data presented are from three separate experiments.

### Animal handling

A total of 60 BALB/c mice of 6 weeks of age and 22–25 g in weight were purchased from the National Laboratory Animal Center (NLAC, Taipei, Taiwan). This study followed the institutional guidelines (Affidavit of Approval of Animal Use Protocol) and was approved by the Institutional Animal Care and Use Committee (IACUC) of China Medical University (Taichung, Taiwan) (13).

### Establishment of the leukemic mice model

A total of 30 BALB/c mice were randomly divided into five groups. Group 1 received an intravenous injection of the solvent (2-glycofurol) as a control, group 2 received an intravenous injection of 1×10^7^ WEHI-3 cells, group 3 received an intravenous injection of CCY-1a-E2 (100 mg/kg/day) for 7 days, group 4 received an intravenous injection of 1×10^7^ WEHI-3 cells as well as CCY-1a-E2 (100 mg/kg/day) for 7 days, and group 5 received an intravenous injection of 1×10^7^ WEHI-3 cells for 7 days and were then administered an intravenous injection of CCY-1a-E2 (100 mg/kg/day) for 7 days. The body weight of each mouse was measured once every 7 days. At day 28, all animals were sacrificed by euthanasia with CO_2_. Blood was collected and spleen and liver samples were obtained and weighed individually as previously described (13,15).

### Immunofluorescent staining

Blood (∼500 *μ*l) was collected from each mouse in different groups and then added to Pharm Lyse lysing buffer (BD Biosciences, San Jose, CA, USA) for lysing of the red blood cells followed by centrifugation for 5 min at 1,500 rpm at 4°C. The isolated leukocytes were examined for cell markers based on being stained with FITC-conjugated anti-mouse CD3, PE-conjugated anti-mouse CD19, PE-conjugated anti-mouse Mac-3 and FITC-conjugated anti-mouse CD11b antibodies (BD Pharmingen Inc., San Diego, CA, USA). Subsequently, cells were analyzed for the levels of specific cell surface markers by flow cytometry as described previously (13,15).

### Safety evaluation

A total of 30 BALB/c mice were randomly divided into five groups. Group 1 received an intravenous injection of PBS as a control. Group 2 received an intravenous injection of the solvent (2-glycofurol) as the solvent control. Group 3 received an intravenous injection of CCY-1a-E2 (5 mg/kg/day) for 7 days. Group 4 received an intravenous injection of CCY-1a-E2 (50 mg/kg/day) for 7 days. Group 5 received an intravenous injection of CCY-1a-E2 (100 mg/kg/day) for 7 days. At day 7, all animals were sacrificed by euthanasia with CO_2_. The body weight, liver weight, spleen weight and biochemical profiles of blood analysis [lactate dehydrogenase (LDH), albumin (ALB), total protein (PRO), serum glutamicpyruvic transaminase (sGPT), serum glutamic-oxaloacetic transaminase (sGOT) and blood urea nitrogen (BUN)] were analyzed as described previously (15–17).

### Statistical analysis

The results are presented as mean ± SEM, and the difference between the CCY-1a-E2-treated and control groups was analyzed by Student’s t-test. P≤0.05 was considered to indicate a statistically significant result.

## Results

### CCY-1a-E2 reduces the percentage of viable WEHI-3 cells

**Figure 2:**
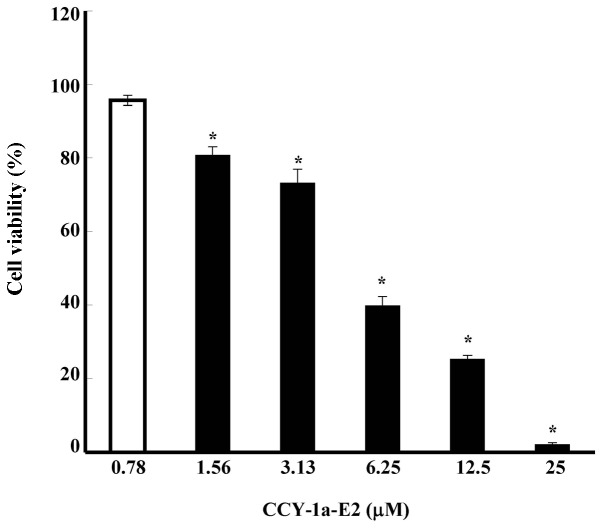
Effects of CCY-1a-E2 on WEHI-3 cell viability. WEHI-3 cells were treated with various concentrations (0.78, 1.56, 3.13, 6.25, 12.5 and 25 *μ*M) of CCY-1a-E2 for 24 h and assessed by MTT assay. Data are presented as mean ± SD of three experiments. ^*^P<0.05, significantly different to dimethyl sulfoxide (DMSO)-treated control.

To evaluate the effect of CCY-1a-E2 on the viability of WEHI-3 cells, we treated WEHI-3 cells with various concentrations of CCY-1a-E2 (0.78, 1.56, 3.13, 6.25, 12.5 and 25 *μ*M) for 24 h. The percentage of viable cells was measured by MTT assay. The results shown in Fig. 2 indicate that CCY-1a-E2 decreased the percentage of viable cells in a concentration-dependent manner for 24 h after the exposure to 0.78–25 *μ*M of CCY-1a-E2 (Fig. 2). The IC_50_ for the 24-h CCY-1a-E2 treatment of WEHI-3 cells was 5 *μ*M.

### WEHI-3 cell allograft model

**Figure 3:**
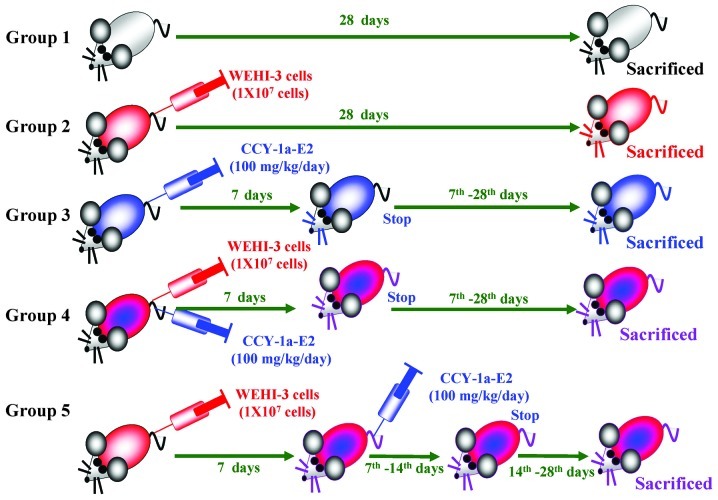
WEHI-3 cell allograft model. Thirty BALB/c mice were randomly divided into five groups. Group 1 received an intravenous injection of solvent (2-glycofurol) treatment as a control, group 2 received an intravenous injection of 1×10^7^ WEHI-3 cells, group 3 received an intravenous injection of CCY-1a-E2 (100 mg/kg/day) for 7 days, group 4 received an intravenous injection of 1×10^7^ WEHI-3 cells and CCY-1a-E2 (100 mg/kg/day) for 7 days and group 5 received an intravenous injection of 1×10^7^ WEHI-3 cells for 7 days and then received an intravenous injection of CCY-1a-E2 (100 mg/kg/day) for 7 days. At day 28, all animals were sacrificed.

**Figure 4:**
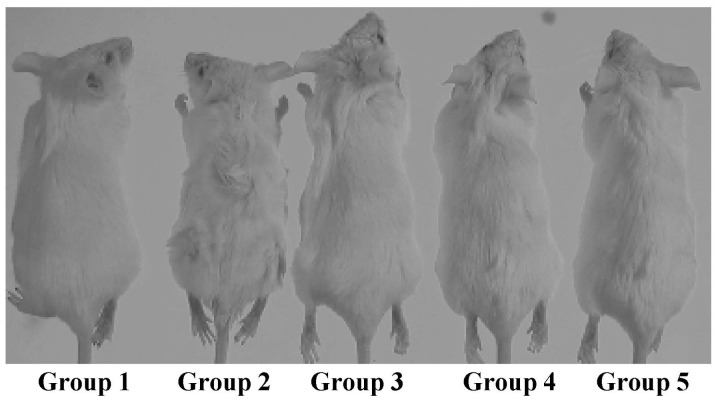
CCY-1a-E2 inhibited leukemia in the WEHI-3 cell allograft model. Representative animals are shown for leukemia formation.

The experimental design and protocol of the leukemic mice model are shown in Fig. 3. Representative mouse images are shown in Fig. 4. At day 28, all animals were sacrificed.

### CCY-1a-E2 reduces leukemia formation in WEHI-3 leukemic BALB/c mice

**Figure 5:**
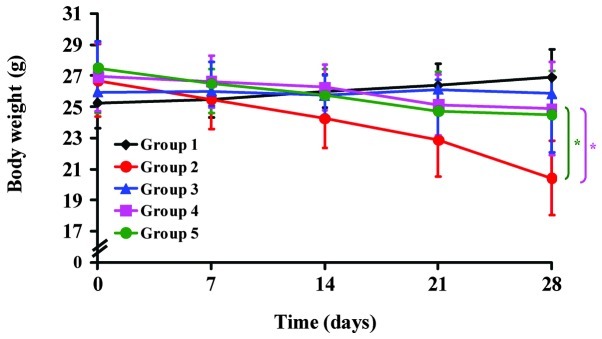
CCY-1a-E2 recovered body weight in the WEHI-3 cell allograft model. Data of body weight from each group are presented as the mean ± SEM of six animals at days 0 to 28 after tumor implantation. ^*^P<0.05 compared with group 2.

**Figure 6:**
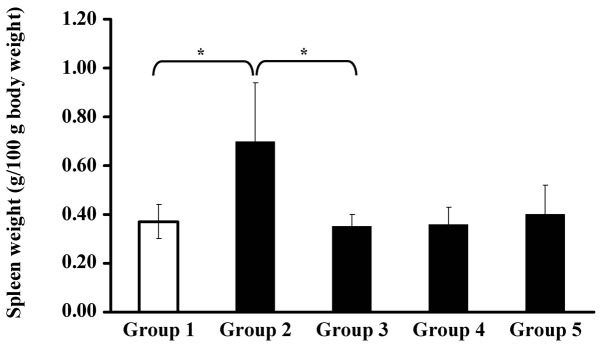
CCY-1a-E2 affected spleen weight in the WEHI-3 cell allograft model. Representative data of spleen weight from each group are presented as the mean ± SEM of six animals at days 0 to 28 after tumor implantation. ^*^P<0.05 compared with group 2.

**Figure 7:**
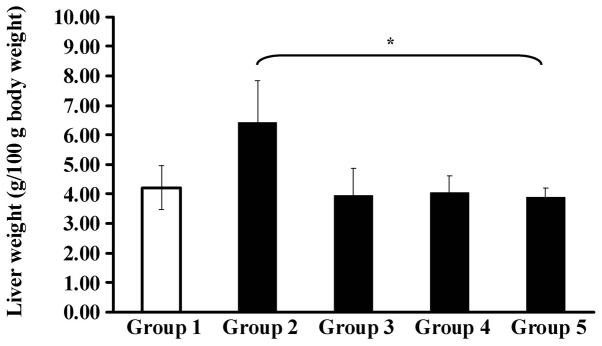
CCY-1a-E2 affected liver weight in the WEHI-3 cell allograft model. Representative data of liver weight from each group are presented as the mean ± SEM of six animals at days 0 to 28 after tumor implantation. ^*^P<0.05 compared with group 2.

We examined the *in vivo* antileukemic activities of CCY-1a-E2 in a BALB/c mouse WEHI-3 allograft model. Representative body weights from the WEHI-3 allograft mice treated with or without CCY-1a-E2 are shown in Fig. 5. The body weight of mice in the CCY-1a-E2 (100 mg/kg) group was not significantly decreased compared with that of the control mice; however, the body weight of mice in the leukemia group was significantly decreased compared with that of the control treatment group. Furthermore, the average body weight in the CCY-1a-E2 (100 mg/kg)-treated leukemic mice increased slightly. As shown in Fig. 6, the spleen weight of group 2 increased significantly; while the spleen weights of groups 3, 4 and 5 did not differ significantly from group 1 (the control group). Fig. 7 shows that the liver weights of the allograft mice were significantly different following treatment with CCY-1a-E2 (100 mg/kg) at day 28.

### CCY-1a-E2 affects surface markers on whole blood cells from WEHI-3 leukemic BALB/c mice

**Figure 8:**
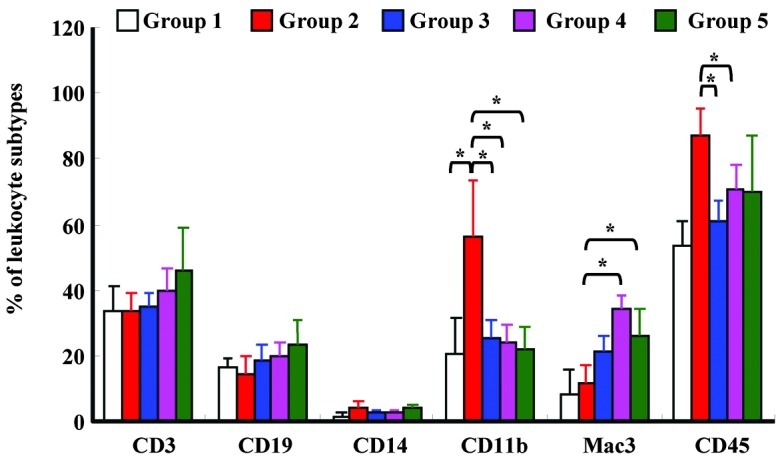
Effects of CCY-1a-E2 on the levels of cell markers in white blood cells from leukemic BALB/c mice. Blood was collected from each animal and analyzed for cell markers (CD3, CD19, CD14, CD11b, Mac-3 and CD45) by flow cytometry as described in Materials and methods. Representative percentages of leukocyte subtypes from each group are shown. Data are presented as the mean ± SEM of six animals at days 0 to 28 after tumor implantation. ^*^P<0.05 compared with group 2.

In order to investigate whether CCY-1a-E2 affects the levels of cell surface markers, leukocytes from CCY-1a-E2-treated and untreated (control) groups were isolated and levels of CD3, CD19, CD14, CD11b, Mac-3 and CD45 were measured. The data from each treatment indicate that CCY-1a-E2 (100 mg/kg) significantly decreased the levels of CD11b and CD45 when compared with the leukemia group (Fig. 8).

### CCY-1a-E2 shows no adverse effects on renal, hepatic and hematological parameters

The safety profile of CCY-1a-E2 (5, 50 and 100 mg/kg) was investigated by pathological examinations and clinical chemistry. Body weight, spleen weight and liver weight were not affected by treatment with CCY-1a-E2 (Table I). The spleen and liver were examined by histopathology. No significant microscopic aberrations were noted compared with the vehicle-treated controls (data not shown). The biochemical measurements of sGPT, sGOT and BUN are shown in Table II. The sGPT and sGOT levels were within the normal value range, suggesting that these groups of mice had normal hepatic function. The BUN assays yielded biochemical values that reflect normal kidney functions. Notably, mice apparently tolerated treatment with CCY-1a-E2, showing no adverse effects on splenic, hepatic or renal parameters.

## Discussion

In a previous study, our groups synthesized several benzyloxybenzaldehyde analogs as novel adenyl cyclase activators and studied the mechanism of action (8). The results showed that 2-benzyloxybenzaldehyde exhibited antiproliferative effects on the vascular smooth muscle cells through the inhibition of the Ras/MAPK signal pathway and its downstream effectors (9). It has also been demonstrated that 2-benzyloxybenzaldehyde inhibits superoxide anion generation through the suppression of Akt and PLD activation in rat neutrophils (10,11). More recently, we have designed and synthesized a new series of 2-benzyloxybenzaldehyde derivatives as anticancer agents (12). In the present study, our results demonstrated that cell viability decreased following treatment with various concentrations of CCY-1a-E2 in a concentration-dependent manner in WEHI-3 leukemia cells (Fig. 2). The IC_50_ of CCY-1a-E2 was 5 *μ*M for the 24-h treatment of WEHI-3 cells. This is in agreement with the previous studies from our investigators, which showed that CCY-1a-E2 decreased the percentage of viable cells in HL-60 cells (12). In addition, CCY-1a-E2 exerts low cytotoxicity on human normal human peripheral blood mononuclear cells (PBMCs; IC_50_ >20 *μ*M). This result shows that CCY-1a-E2 was less toxic for PBMCs than for WEHI-3 cells.

*In vivo* model systems of leukemia were established for the evaluation of new antileukemic agents (15,18). The murine allograft model is frequently used for experimental antileukemic therapy as it is quick and easy to induce leukemia (13,19). The murine WEHI-3 myelomonocytic leukemia cell line originally derived from the BALB/c mouse was first established in 1969 and it has been used to induce leukemia in BALB/c mice for evaluating the antileukemic effects of drugs (13,20,21). There is no information concerning the effects of CCY-1a-E2 on leukemia cells *in vivo*. In the present study, the antileukemic effects of CCY-1a-E2 on WEHI-3 leukemia cells were first investigated *in vivo*. Our results suggest that CCY-1a-E2 had a growth inhibitory effect in WEHI-3 cells *in vitro* and also affected leukemia formation *in vivo.* In addition, treatment with CCY-1a-E2 significantly inhibited the loss of body weight compared with the leukemia group (Fig. 5). CCY-1a-E2 also inhibited the spleen and liver growth of leukemic mice (Figs. 6 and 7). The results of histopathological examination indicate that the infiltration of immature myeloblastic cells into the splenic red pulp in spleen sections was eliminated in CCY-1a-E2-treated leukemic mice when compared with the untreated leukemia group (data not shown). Moreover, CCY-1a-E2 reduced the levels of CD11b (monocytic marker) in comparison to the leukemia group. Therefore, intraperitoneal administration with CCY-1a-E2 to leukemic mice altered the specific surface markers from PBMCs *in vivo*. Our results show that CCY-1a-E2 is a promising candidate as an antileukemic agent and our studies provide useful information for the development of a new therapeutic strategy against leukemia.

In conclusion, our study is the first to demonstrate that CCY-1a-E2 has growth inhibitory effects in WEHI-3 leukemia cells. *In vivo* results indicate that CCY-1a-E2 has no adverse effects on renal, hepatic or hematological parameters and exerts the ability of antileukemic activity in WEHI-3 allograft model of leukemia.

## Figures and Tables

**Table I:** Change in body, spleen and liver weight in the BALB/c mice following treatment with CCY-1a-E2 by intravenous injection once every day for 7 days.

Item	Control	2-glycofurol (solvent control)	CCY-1a-E2 (5 mg/kg)	CCY-1a-E2 (50 mg/kg)	CCY-1a-E2 (100 mg/kg)
Body weight (g)	24.58±0.66	28.20±1.10^a^	28.42±1.13^a^	28.88±1.52^a^	28.90±0.51^a^
Liver weight (g/100 g body weight)	4.43±0.47	3.98±0.30	4.42±0.47	4.18±0.33	4.17±0.26
Spleen weight (g/100 g body weight)	0.33±0.05	0.28±0.04	0.35±0.06	0.31±0.06	0.37±0.04

Values are presented as mean ± SD (n=6);

* P<0.05 compared with the control group.

**Table II:** Biochemical profiles of blood analysis in the BALB/c mice following treatment with CCY-1a-E2 by intravenous injection once every day for 7 days.

Item	Control	2-glycofurol (solvent control)	CCY-1a-E2 (5 mg/kg)	CCY-1a-E2 (50 mg/kg)	CCY-1a-E2 (100 mg/kg)
LDH (U/l)	255.50±105.18	327.00±162.48	298.00±62.22	244.83±46.99	270.83±42.09
ALB (g/dl)	1.63±0.28	1.71±0.08	1.58±0.38	1.73±0.26	1.84±0.13
PRO (g/dl)	3.81±0.69	3.90±0.95	3.68±0.60	4.06±0.58	4.77±0.46
sGPT (U/l)	38.25±7.49	38.58±10.31	41.83±8.94	43.92±16.02	41.83±10.02
sGOT (U/l)	85.58±12.24	92.25±37.19	105.83±22.16	79.98±26.10	115.12±15.08
BUN (mg/dl)	26.83±2.09	27.58±1.80	30.33±5.02	26.17±2.42	28.92±2.20

LDH, lactate dehydrogenase; ALB, albumin; PRO, total protein; sGPT, serum glutamic-pyruvic transaminase; sGOT, serum glutamic-oxaloacetic transaminase; BUN, blood urea nitrogen. Values are presented as mean ± SD (n=6).
